# Supplementary figures and images for: Acquisition of ionic copper by the bacterial outer membrane protein OprC through a novel binding site
Source: PLoS Biol. 2021 Nov 11;19(11):e3001446. doi: 10.1371/journal.pbio.3001446 (PMC8610252; doi:10.1371/journal.pbio.3001446)

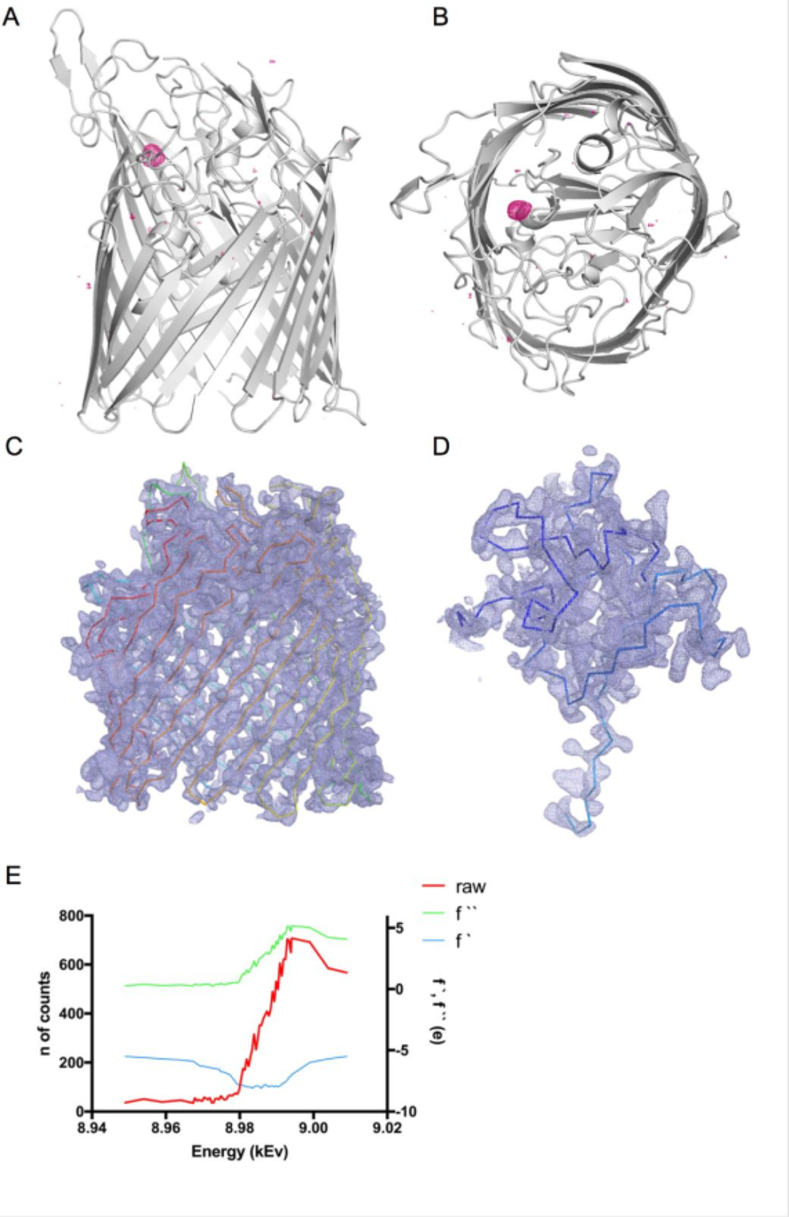

Supplement: S1 Fig — (A, B) Copper anomalous maps (coloured magenta) contoured at 4 σ (carve = 30). Experimental density for one OprC protomer after density modification (but before model building) for (C) barrel and (D) N-terminal plug domain (map contoured at 1.5 σ, carve = 2.0). Ribbon is shown for orientation purposes. (E) X-ray fluorescence spectrum showing the copper-specific energy peak. Underlying data for panel E can be found in S1 Data. (TIF) [file pbio.3001446.s001.tif]

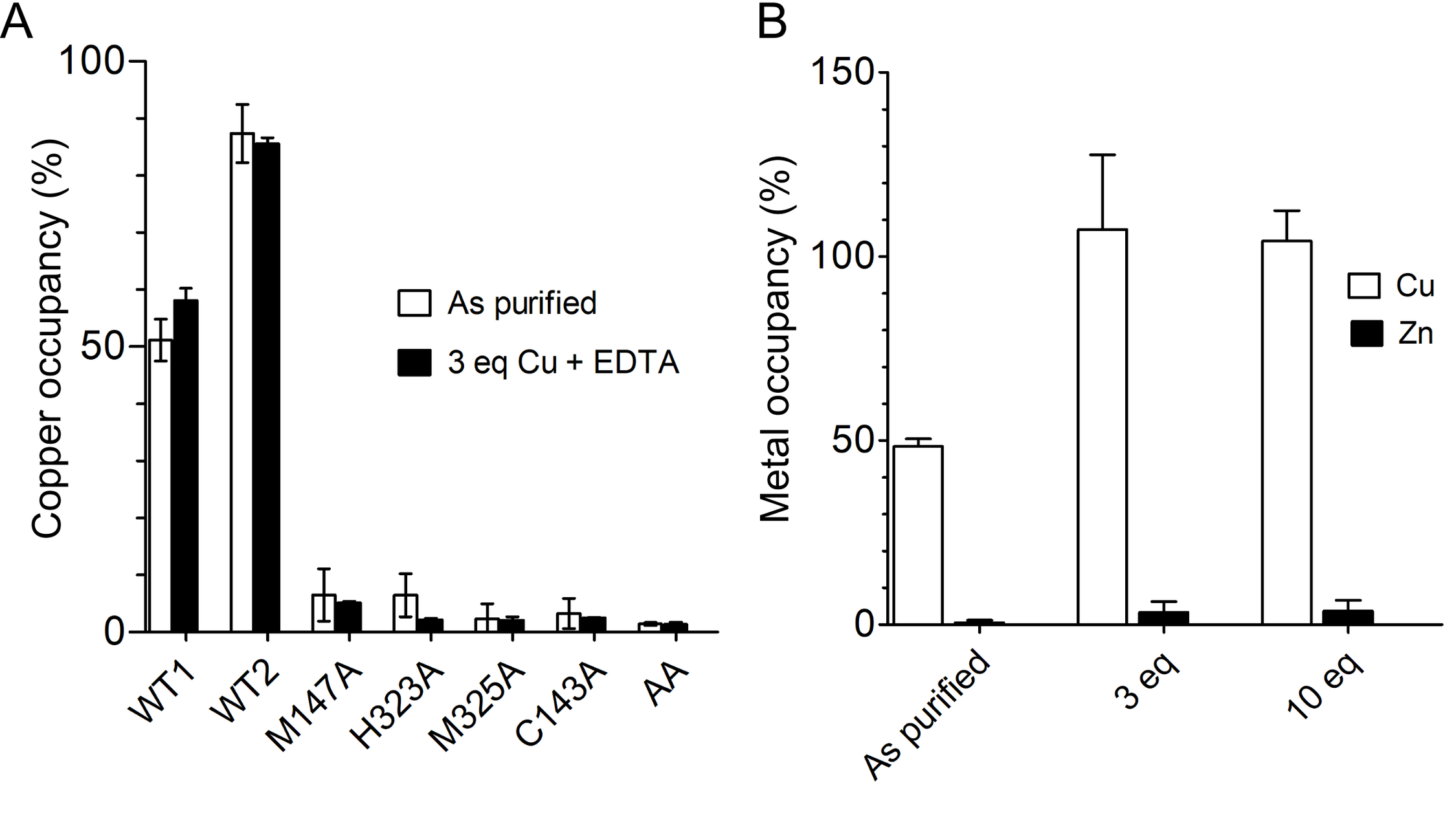

Supplement: S2 Fig — (A) Metal occupancy of OprC and mutant proteins after incubation with 3 equivalents copper in the presence of 0.5 mM EDTA (approximately 50-fold excess) followed by analytical SEC and subsequent metal analysis by ICP-MS. (B) Metal occupancy of OprC WT after incubation with 3 or 10 Eq. of Cu or Zn. Underlying data for this figure can be found in S1 Data. EDTA, ethylenediamine tetra-acetic acid; ICP-MS, inductively coupled plasma mass spectrometry; SEC, size exclusion chromatography; WT, wild-type. (TIF) [file pbio.3001446.s002.tif]

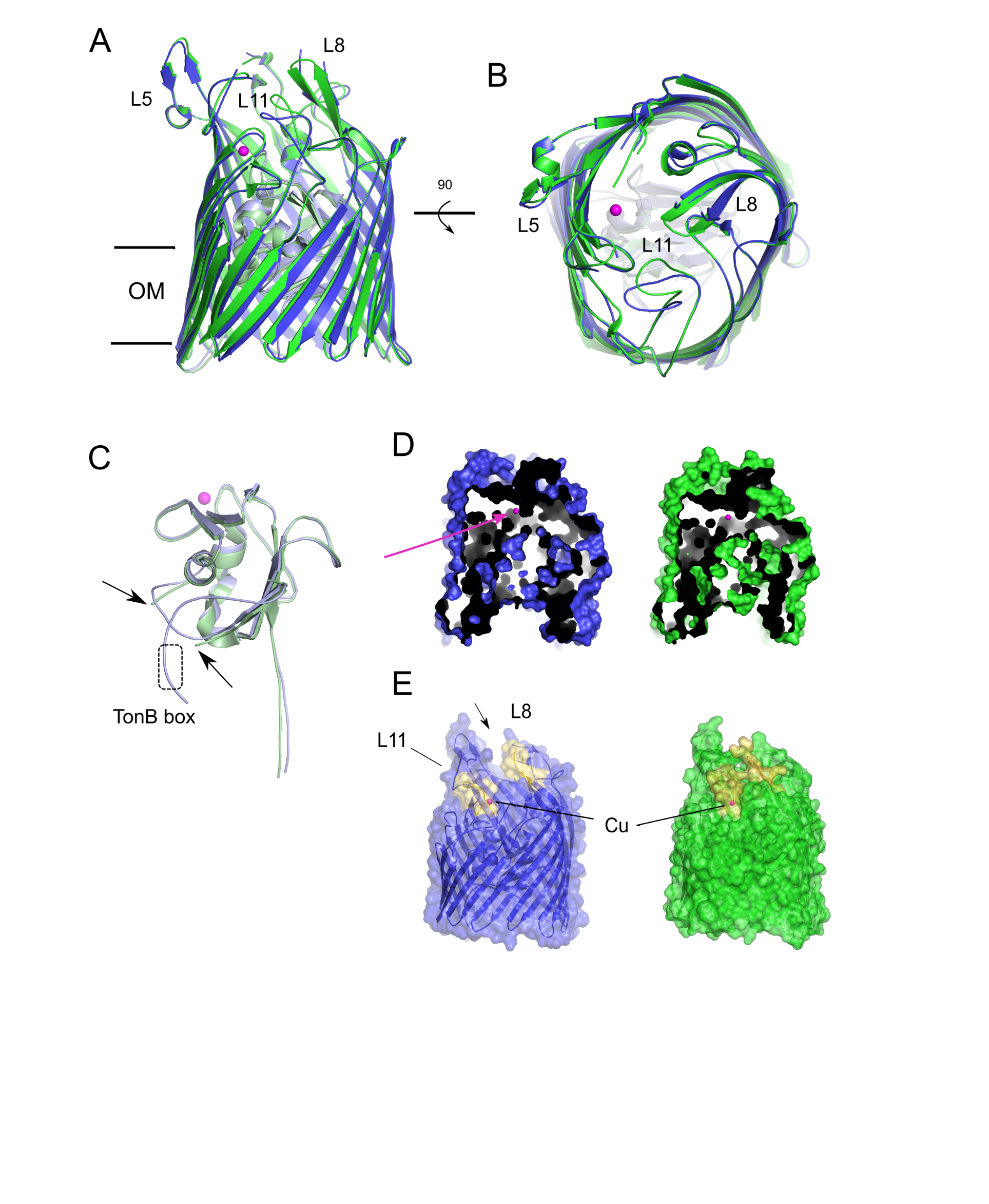

Supplement: S3 Fig — (A, B) Cartoon superposition from the OM plane (A) and extracellular environment (B) of OprCWT (coloured green) and OprCAA (blue), indicating locations of loops L5, L8, and L11; copper is represented as a magenta sphere. The plug domains of OprCWT and OprCAA are coloured light green and light blue, respectively. (C) Superposition of N-terminal plug domains indicating the location of the TonB box, which is invisible in Cu-bound OprCWT. Arrows indicate the missing density for Glu88-Pro94 in OprCWT. (D) Surface slab representations from the OM plane, showing the presence of a solvent pocket in OprCAA that is generated by the absence of Met147 (arrow). For orientation purposes, the OprCWT-bound copper is shown in both structures. (E) Side surface views showing the conformational changes of L8 and L11 (coloured yellow) as a result of copper binding. As in (D), the bound copper of OprCWT is shown in both proteins. OM, outer membrane; TBDT, TonB-dependent transporter; WT, wild-type. (TIF) [file pbio.3001446.s003.tif]

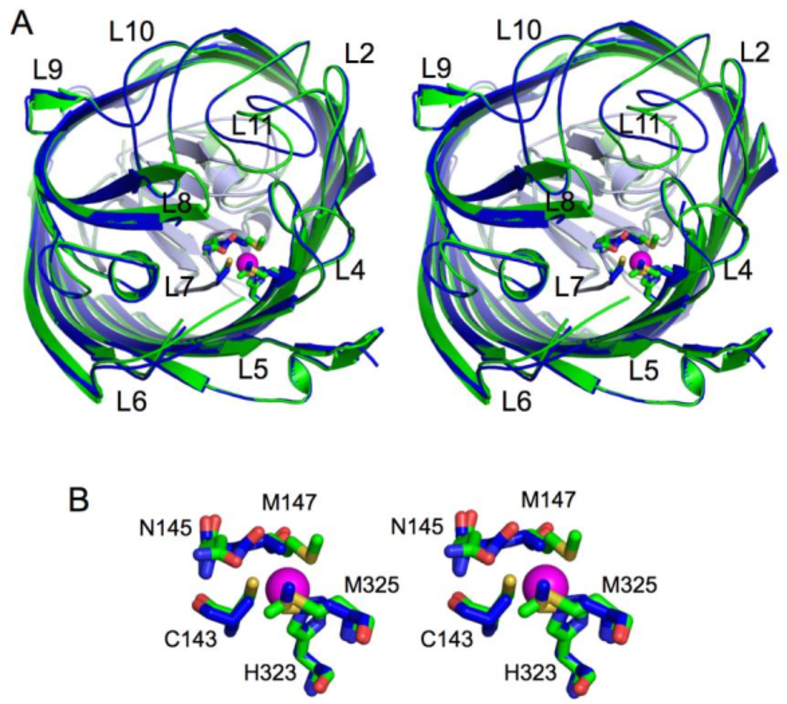

Supplement: S4 Fig — Stereo 3D representation of superposed OprC (coloured green) and OprCAA (blue). (A) Extracellular view showing loops 2, 4, 5, 6, 7, 8, 9, 10, 11 (L2, L4, L5, L6, L7, L8, L9, L10, L11). Conformational changes are observed for external loops L8 and L11. (B) Active site view illustrating superposed residues involved in metal coordination for WT OprC (green sticks) and OprCAA (blue sticks). Asn145 (N145) is also shown due to its role in shielding the active site. Oxygen atoms in amino acid residues are coloured red, nitrogens blue, and sulphurs yellow. The copper atom is represented as a magenta sphere. WT, wild-type. (TIF) [file pbio.3001446.s004.tif]

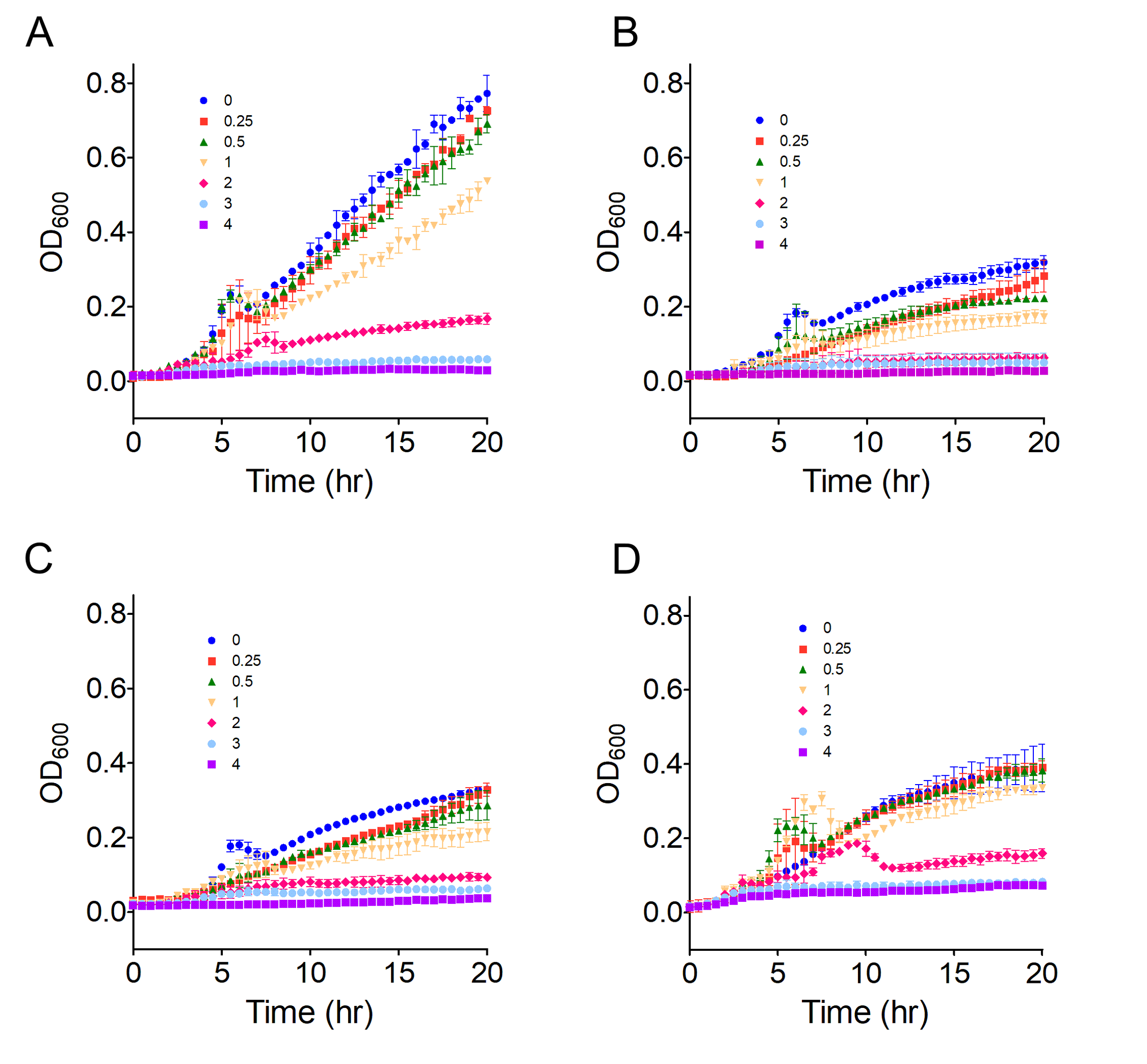

Supplement: S5 Fig — Anaerobic growth of (A) pHERD30 (empty plasmid) and pHERD30-overexpressed (B) OprCWT, (C) OprCC143A, and (D) OprCAA in PA14 ΔoprC was monitored during copper stress in rich media supplemented with 100 mM sodium nitrate. Overexpression was induced with 0.1% arabinose. Values indicate externally added copper in mM. Underlying data for this figure can be found in S1 Data. (TIF) [file pbio.3001446.s005.tif]

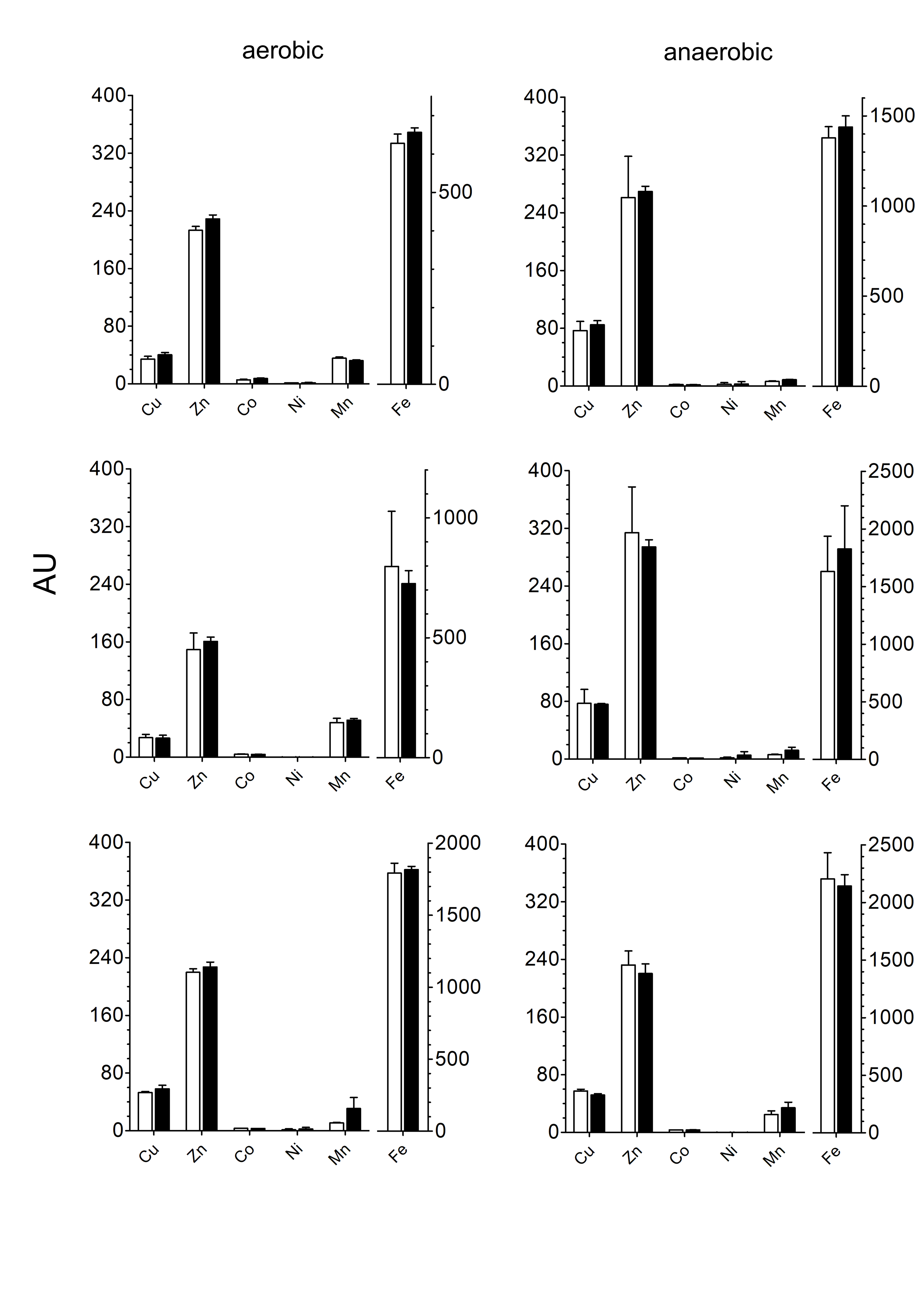

Supplement: S6 Fig — Cell-associated metal content was determined in cells grown in rich media supplemented with 100 mM sodium nitrate under both aerobic (left panels) and anaerobic conditions (right panels) without added copper. The 3 biological replicates have been plotted separately due to the different absolute metal contents. The right y-axis is for Fe content. Reported values are averages ± SD (n = 3). Underlying data for this figure can be found in S1 Data. ICP-MS, inductively coupled plasma mass spectrometry; WT, wild-type. (TIF) [file pbio.3001446.s006.tif]

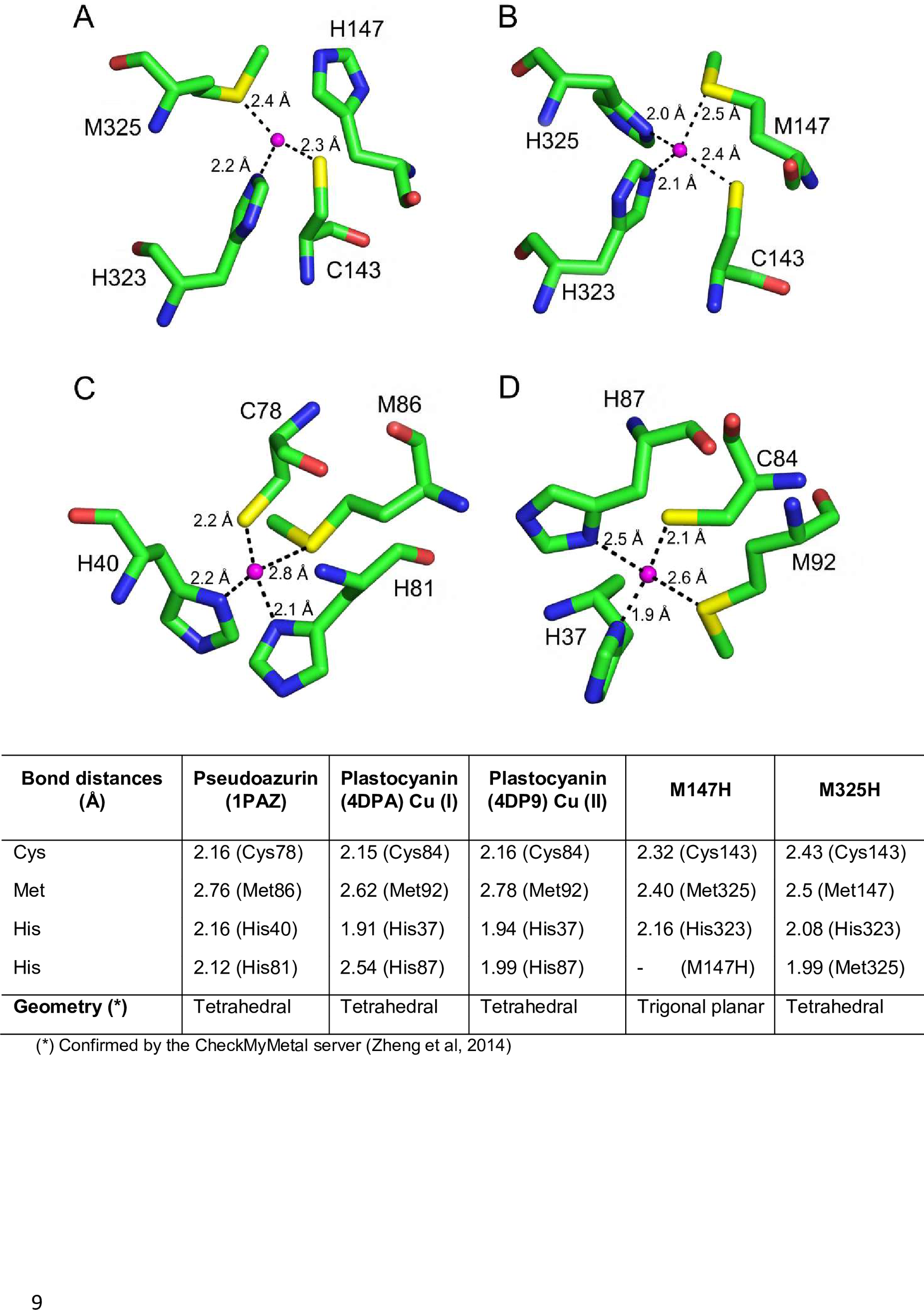

Supplement: S8 Fig — Close-up views of copper binding site residues in (A) M147H, (B) M325H, (C) pseudoazurin (PDB ID 1PAZ), and (D) plastocyanin (PDB ID 4DPA). The bound form of copper is Cu(I). Distances between coordinating residues and metal (magenta) are shown. The table summarises distances between copper and coordinating residues as well as geometry. (TIF) [file pbio.3001446.s008.tif]

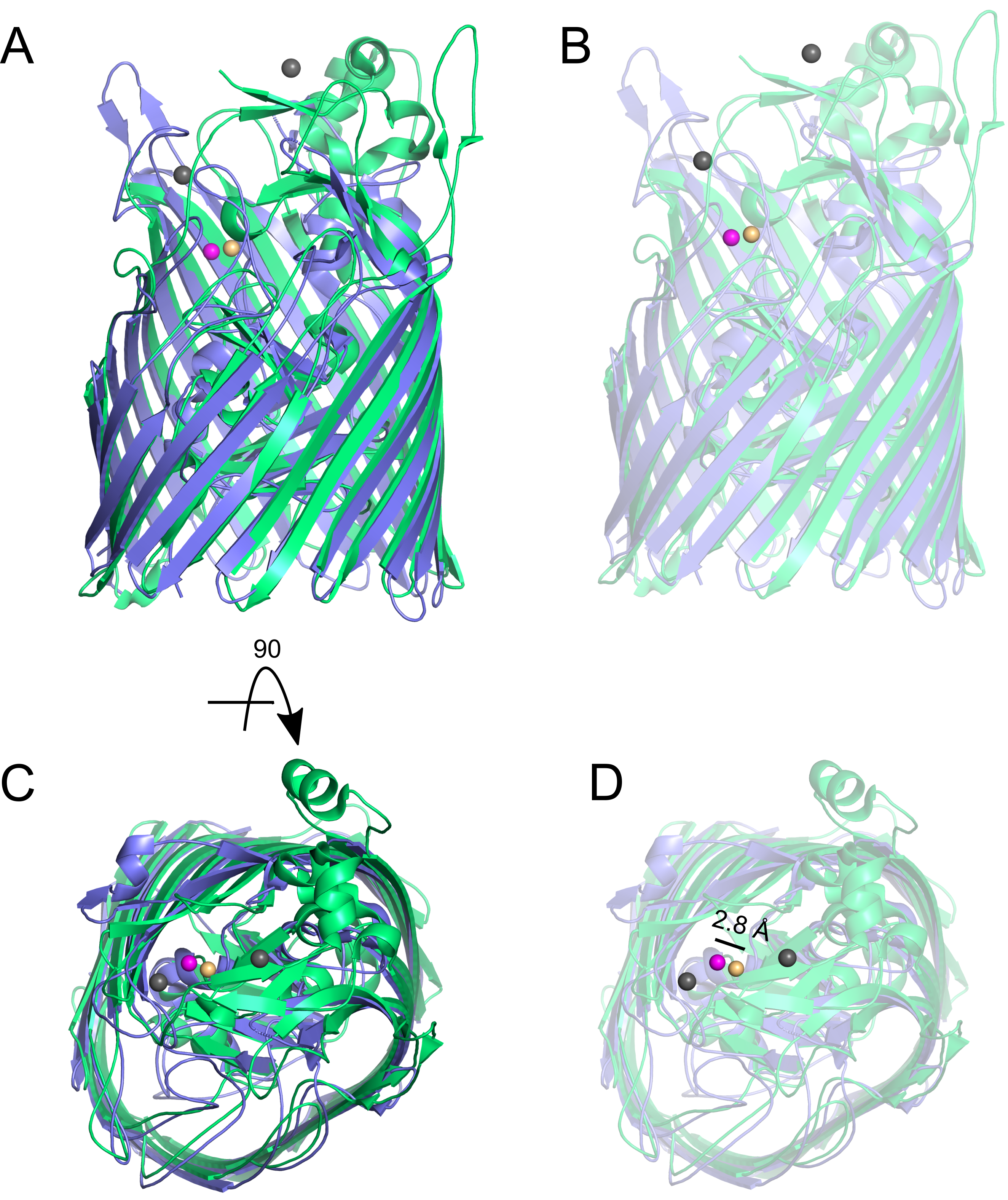

Supplement: S9 Fig — (A, C) Cartoon representation comparing Cu-loaded OprC (coloured blue, copper atom shown as magenta sphere) and the locked version of ZnuD (coloured green, 2 cadmium atoms bound to low-affinity sites represented as grey spheres, zinc bound to the high-affinity site represented in orange; PDB ID 4RDR). The locked conformation of ZnuD shows low-affinity metal sites at external loops, at regions similar to the methionine track (L5) from OprC. (B, D) Transparent view of the secondary structures illustrates the similar topological location for the high-affinity metal sites (distance of 2.8 Å). (TIF) [file pbio.3001446.s009.tif]

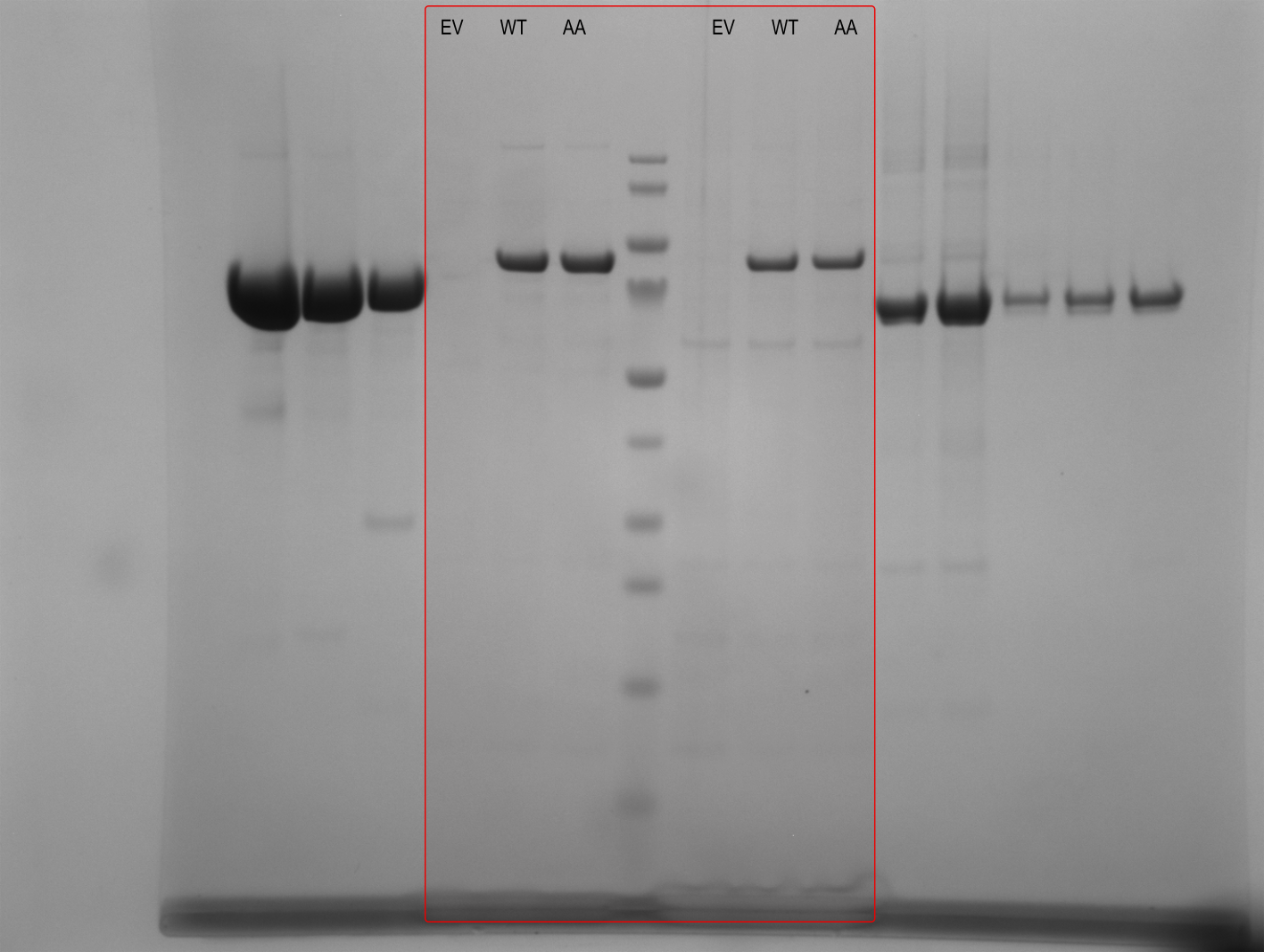

Supplement: S2 Data — (TIF) [file pbio.3001446.s014.tif]
