# Supplementary material for: Acquisition of ionic copper by the bacterial outer membrane protein OprC through a novel binding site
Source: PLoS Biol. 2021 Nov 11;19(11):e3001446. doi: 10.1371/journal.pbio.3001446 (PMC8610252; doi:10.1371/journal.pbio.3001446)
Supplement: S7 Fig — Shown are P. aeruginosa (uniprot ID G3XD89), NosA from P. stutzeri (uniprot ID Q00620), Pseudomonas putida (uniprot ID Q88DI7), Pseudomonas syringae (uniprot ID A0A085VGG7), A. baumannii (uniprot ID A0A0G4QL30), S. enterica (uniprot ID A0A505CFK3), K. pneumonia (uniprot ID A0A486MDQ0), Serratia marcescens (uniprot ID A0A221DQ80), and Enterobacter cloacae (uniprot ID A0A1S6XXV6), showing high conservation of the binding site residues Cys143 (highlighted in yellow), Met147 and Met325 (green), and His323 (cyan). Methionine track residues are depicted in red, and those located in the N-terminal plug are coloured magenta. The TonB box sequence is depicted in blue. The zinc transporter ZnuD from Neisseria meningitides (uniprot ID Q9JZN9) is shown for comparison. Numbering is for the full-length P. aeruginosa OprC sequence. Clustal scoring is indicated below the alignment. (PDF) [file pbio.3001446.s007.pdf]

```
                     53   |          |           |                |         100     |         |
P.aeruginosa   ----AEEHSQHQDHAV-ELAPSVVTGVA---QSSPLTI-------VTNPKEPRQPVPASDGADYLKTIPGFAVIRNGGSNGDPVLR
P.stutzeri     AESVDHSEHAHHASSA-ELAPMVITGVA---QQSPLTV-------ATDPKIPRQPVPASDAGDYLQTIPGFSAVRGGGSNSDPVFR
P.putida       ---AGPGHEDHVHDAP-ELSPTVITAVA---PSSPLTV-------VTNPKDPRQPVPASDGADYLKTIPGFSAIRAGGTNGDPVLR
P.syringae     -AQPQDDETDTQQPTL-GLSPLVITAVQ---QSSPLTV-------VTNPKDARQPVPASDGTDYLKTIPGFSSIRSGGSNGEPVLR
A.baumannii    ----ESEKNDAETNTLHSLAPIVVTAQQG-NDANGLIV-------HADPKQPIQPVPASDGADYLQSIMGFNSIQSGGTNGDVTFR
S.enterica     ------ATVKNQNIAKDTDADVITVTAP---VTSPLEI-------ITSPKEPRQPVPASDGSDYLKTIPGFSQIRNGGTNGDPVFR
K.Pneumoniae   -------ARESHDYATMEDDSVMVVTAP---ASSPLEV-------VTSPKRPRQPVPASDGSDYLKTIPGFSQIRAGGTNGDPVFR
S.marcescens   --------HQHPTDAQVNDGDVITVTAP---LYSPLTI-------VTSPKTPRQPVPASDGSDYLKTIPGFSQIRNGGTNGDPVFR
E.cloacae      --------QESHDHATMEDDSVMVVTAP---ALSPLEV-------VTSPKRPRQPVPASDGSDYLKTIPGFSQIRAGGTNGDPVFR
ZnuD           ----------HETEQSVDLETVSVVGKSRPRATSGLLHTSTASDKIISGDTL--RQKAVNLGDALDGVPGIHASQYGGGASAPVIR
                                       .     . *          . .         * :   * *. : *:    : **   .  .:*

                     |         |    150    |         |          |         |    200
P.aeruginosa   GMFGSRLNILTNGGMMLGACPNRMDAPTSYISPETYDKLTVIKGPQTVLWGPGASAGTILFER--EPERFG-ELGSRVNASLLAGS
P.stutzeri     GMFGSRLKLLANGAEMLGACPSRMDSPSSYITPENYDALTVIKGPQTVLWGPGNSAATILLER--DPEDFS-ELGGRIDASFLVGS
P.putida       GMFGSRLNILTNGGLMLGACPNRMDAPTSYISPETYDRLTVIKGPQSVIWGPGGSAGTILFER--EPEKFG-TLGSRVNASLLAGS
P.syringae     GMFGSRLNILRTNGGLMLGACPFRMDAPTSYIAPETFDKLTVVKGPQTVQWGPGASAGTVLFER--EPEHFG-ELGSRLNGSVLAGS
A.baumannii    GMFGSRIKILTDGTENLGACPNRMDAPTSYISPESYDRISVIKGPQTVQYANTGSAATVLFER--QPEKLTSEKPYRGQASVLLGS
S.enterica     GMFGSRLKILTDGAEMLGACPSRMDAPTSYIAPEDFDLLSLIKGPETVLWGPGNSAGTIRFDR--ETPSFE-TNAVKGTASVLAGS
K.Pneumoniae   GMFGSRLRILTNNGEMLGACPARMDAPSSYISPESFDLLTLTKGPQTVLWGPGNSAGTIRFDR--EQPRFN-KPGVQGNASLLAAS
S.marcescens   GMFGSRLKILTDGSEMLGACPSRMDAPTSYISPESFDLLTITKGPQTVLWGPGSSAGTVRFER--ERPRFD-KPGIKGSASVLTGS
E.cloacae      GMFGSRLRILTNNGEMLGACPARMDAPSSYISPESFDLLTLTKGPQTVLWGPGNSAGTIRFDR--EQPRFD-KPGVQGNASLLAAS
ZnuD           GQTGRRIKVLNHHGETGDMADFSPDH-AIMVDTALSQQVEILRGPVTLLYSSGNVAGLVDVADGKIPEKMP-ENGVSGELGLRLS-
               *   * *:.:    .      .  .     *    :    :           : : : :** :: :.      *. : .              :         ..

                  |          |          |         |        250          |         |         |
P.aeruginosa   NGRFDKVLDA--AAGNRLGYL-RFTGNHAQSDDYEDGAG---NTV-PSRWKKWNGDVAVGWTPDEDTLIELTAGKGDGEARYAGRG
P.stutzeri     DGRFDRNIDA--AAGGEQGYI-RLLANRSDSDDYQDGNG---DDV-HSRWDKWSTDLVLGWTPDEDTLLELTVGRGDGEARYAGRM
P.putida       NGRFDKVLDA--AAGNSQGYA-RFVGNQSRSDDYHDGNK---DTV-PSRWEKWNGDVALGWTPDQDTLIELTAGKGDGEARYAGRG
P.syringae     NGRFDKVLDG--AVGGPEGYM-RVVGNQAQADDYKDGRG---NTV-PSRWEKWNGDVALGWTPDADTLIELTAGKGNGEARLGGRG
A.baumannii    YGRIDHNIEA--AVGDEKKYI-RLNANRSESNSYQDGDG---NTV-PSAWKKWNVDVALGFTPDENTWVEITGGKSDGESLYAGRS
S.enterica     RDRYDGNADI--SLGSEKGYL-RLTGNKSRSSDYKDGNG---KNV-HSGWDKWNSDITVGITPEADRLIEFSAGTGNAQAAYAGRA
K.Pneumoniae   NNRWDENADI--SLGSEDGYL-RLMGNKSRSDDYKDGNG---DRV-PSKWDKWNGDMALGWTPDKDTLIELTAGKGDGESRYAGRS
S.marcescens   NGRWDENIDA--SLGAEQGYL-RVMANKSRSNDYQDGTN---TRV-PSRWDKWNGDLALGWTPDNDTLLEVTMGRGNGEARYAGRS
E.cloacae      KNRWDENADI--SLGSEDGYL-RLMGNKSRSDDYKDGNG---DRV-PSKWDKWNGDMALGWTPDKDTLIELTAGKGDGESRYAGRS
ZnuD           SGNLEKKLTSGGINIGLGKNFVLHTEGLYRKSGDYAVPRYRNLKRLPDSHADSQTGSIGLSWVGEKGFIGVAYS---DRRDQYG-LP
                 .. :     .        *       :     :    .      :..*                 :    *    .. .  . .: :. .  :  .                   :  .     .

                  |         300            |         |        |
P.aeruginosa   MDGSQFKRESLGLRFVKSNVSDVLEKVEAQVYYNYADHIMDNFRLRTPDPS-----------------------------------
P.stutzeri     MDGSQFERESVALRFEKTNLGENLKKIEARVYYNYADHVMDNYSLRTPPM------------------------------------
P.putida       MDGSQFKRESLGLRFEKSNLGEVLDKVEAQVYYNYADHVMDNYSLRTPSGS-----------------------------------
P.syringae     MDSSQLERESLGLKFEKRNLGGVLDKLEAQVYYNYADHIMDNFRLRTPDPA-----------------------------------
A.baumannii    MDGSQFARESLGLRFEKKNITDVIKKIEGQVNYSYNDHVMDNFSLREFNPQ-----------------------------------
S.enterica     MDGTEFKRQSLGMHFVFSDLGSVFDKFEGQINYNYARHVMDNYSLRQLPQN-TGDHGMH-MM------------------------
K.Pneumoniae   MDGSQFRRESLGARFEKSNIGEVFQKFEANVYYNYADHIMDNYSLRSPDGGMSGGMSEG-MT------------------------
S.marcescens   MDGSQFKRESLGMRVEKSNIGEVLDKLEAQVYYNYANHVMDNVTLRSPGSGGMGGHGGH-G-------------------------
E.cloacae      MDGSQFSRESLGARFGKSNIGEVFQKFEANVYYNYADHIMDNYSLRSPGGGMSGGMSEG-MA------------------------
ZnuD           AHSHEYDDCHADIIWQKSLINK--------RYLQLYPHLLTEEDIDYDNPGLSCGFHDDDNAHAHTHSGRPWIDLRNKRYELRAEW
                 .. :                 :                 .      *:: :                                 :

                                         |       350      |         |          |         |
P.aeruginosa   --------------------------SMMP------MPMASQVDRRTLGGRLAATWRWDDFKLVTGVDAMRNEHRARGSKYDMMTD
P.stutzeri     ----------------------------------MPQMATNVDRRTLGGRMAATWQLDEYELVTGVDAQTNEHRRRGGV------
P.putida       --------------------------GMMG------MPMVSNVDRRTMGARIKATWRWADVQLISGIDAQTNEHRQRGGMGV----
P.syringae     --------------------------SMMA------MPMASQVDRRTVGGRVAATWKWQDVELVTGVDALRSEHRERNSTYDMMTD
A.baumannii    --------------------------DGMS------MPMASNVARRTLNARLAMTNEWSQWSFISGVDTQNNKHSSRSSMRS----
S.enterica     ------------HAD-SGSMHH-MQGMKMG------GKMIMPYDRRTVSGRLMGTWDWEDVKLEAGTDTQMYTHRSVKMYNP----
K.Pneumoniae   ------------DSGMGDSMDA-GMSMDMN------MPMAMEVDRRTVGGRMMGTWEWADVELKSGADTQLNIHRNK---------
S.marcescens   ------------AMSMG----G-HGGHMMS------SGMTMQLDRRTVGGRVMGTWQWQDVKLESGLDTQTNTHRSM---------
E.cloacae      ------------ESGMGDSMDA-GMSMDMN------MPMAMEVDRRTVGGRMMGTWEWADVELKSGADTQLNTHRNK---------
ZnuD           KQPFPGFEALRVHLNRNDYRHDEKAGDAVENFFNNQTQNARIELRHQPIGRLKGSWGVQYLQQKSSALSAI---------------
                                                      *:    .*:   :       .   :.   :

                    400       |         |          |          |         450     |         |
P.aeruginosa   YYTDADQFPWSKDAVFHNYGAFGELTWFAAERDRLIGGLRLDRASVKD-YRQTLKSG--HMGHAMANPTANDTRADTLPSGFVRYE
P.stutzeri     ---DYKSKPWEKDADFHNYGLFGELTRTLNDDSRVIGGARLDHATAKD-YRSTG-------------PSAGDSRSDNLPSGFLRYE
P.putida       --DAHKGKAWTKDADFHNYGAFSELTWYVSGEDRLITGARLDRASARD-FRTTS-------------ATEGDTRADTLPSGFIRYE
P.syringae     IYTDTDAFAWSKDAVEHNYGAFAEMTWYAAERSRIVSGARLDRASAKD-YRQAITSM----SMSVPNPTANETRADTLPSGFARYE
A.baumannii    ---NYLNQPRVTDMIFHSYGAFGELGYQWNDFNKLVTGVRLDRVTVED-ERAKSKDF-------------NTKLEKTLPSAFVRWE
S.enterica     --DTSGAGPWNKDARFHDYGIFAQTTWNINNDYDLITGARIDHAQMKS-FKKA-----------------ERKRDAYLPAGFVRTE
K.Pneumoniae   -----MENSWVKDARFHDYGLFSELTWNTSDSSKLVGGARLDRVLVDN-FSGKG----------------SSERTDTLPAGFVRFE
S.marcescens   -----SRGSWEKDAQFNSYGAFSELTWSTSEQDKLIGGARLDRTLVEN-FRSGS----------------DGERSDTLPSGFMRLE
E.cloacae      -----MDNSWVKDARFHDYGLFSELTWNTSDSSKLVGGARLDRVLVDN-FSGKG----------------SSERTDTLPAGFVRFE
ZnuD           --SEAVKQPMLLDNKVQHYSFFGVEQANWDN-FTLEGGVRVEKQKASIQYDKALIDRENYNHPLPDL--GAHRQT--ARSFALSG
                               *     : *. *.            :   *  *:::                                         .*

                      |          |        500        |              |          |          |    550
P.aeruginosa   HDLADSPTTLYAGLGHAERFPDYWELFSPKRGPNGSVNAFD----KIKPEKTTQLDFGLQYNGDKLQAWASGYVGVVQDFILFSYR
P.stutzeri     HDLQSLPATAYVGLGHTQRFPDYWELFSGG------ADAFE----KLDPEKTTQLDFGLQYSKGPLDAWVSAYVGQVRDYILFSYS
P.putida       HDLAAIPATTYIGLGHAQRFPDYWELFSPKLAPPGAANAFD----GIKPEKTTQLDFGIQYRTERLEAWASGYVGQIRDYILFDYR
P.syringae     YDLADSPTTLYAGIGHVQRFPDYWELFSGGSGPAGSRNAFE----GVKPEKTTQLDFGAQFNGEDLQAWVSGYVGQVRDFILFDYS
A.baumannii    NQHPEHELKSYIGLGYVERMPDYWELFSPIHGNAGSTNTFN----GVNPEKTTQLQLDMGFQQHGALSTWASAYAGLVDDYILMSYH
S.enterica     HTFSDKKGMMYAGLGYVKRFPDYWELFSSTNSKYGLEDAFT----SVRPEETTQLDIGTQYNIGDVTTWVSFYTAYINNYIIFQYD
K.Pneumoniae   HTLAEMPLMLYAGLGYTERFPDYWELFSPTYGPDGTLDAFD----KVKTEKTTQLDIGAQYSGKRTNAWVSAYVGRVNDFILFRYD
S.marcescens   HTLADLPLMLYAGVGYTERFPDYWELFSPKLGPNGSKDPFS----SVKSEKTTQLDIGAQYNGKRFNGWVSAYVGRVDDFILFKYD
E.cloacae      HTLAEMQLMLYAGLGYTERFPDYWELFSPTFGPDGTSDAFD----KVKTEKTTQLDIGAQYSGKRTNAWVSAYVGRVNDFILFRYD
ZnuD           NWYFTPQHKLSLTASHQERLPSTQELYAHGKH--VATNTFEVGNKHLNKERSNNIELALGYEGDRWQYNLALYRNRFGNYIYAQTL
                                   .: :*:*.   **:                 :  *          :    *.:  ::::.                 :  *     .  ::*

                              |          |          |         |      600      |
P.aeruginosa   EGM---------MGSST-QATNVDARIMGGELGASYQLTGNWKTDASLAYAWGKNSSDDRALPQIPPLEARFG-------------
P.stutzeri     PS---------------KYSENIDARIMGGELGATYRLTSNWKTDASLAYAWGKNSSDGEALPQMPPLEGRLG-------------
P.putida       TGM---------MGMSTSQAQNIDARIMGGELGAAYQLTDNWKADATLAYAWGKNSSDGKALPQMPPLESRLG-------------
P.syringae     TDM---------MGMSSSRTENVDARIMGGELGFAYRLSPNWKTDATLAYAWGKNSSDGQALPQIPPLEGKLG-------------
```

```
A.baumannii         HHPSMGMDGHGMSHGITAGAKNVDATIAGAEAGIGYQFTDHIQADLSAMYAWGKNTTDDKPLPQISPLEGRLN-------------
S.enterica          PSD---------KKGKTSKAYNVRARTLGAESGLSWQFIPDWKFDTSLAWSWGQNITEDQPLPQMPPLEGRFA-------------
K.Pneumoniae        PND---------AY--ISQVDNINATIMGGEAGVSYKLTDSWKTDASLAYSWGRNTENGKPLPQMPPLEARLG-------------
S.marcescens        PHN---------AR--LSQADNVNANIMGGEMGMGYQLSEHWKTDASLAYSWGKNTSDGRPLPQIPPLEARLG-------------
E.cloacae           PND---------AY--ISQVDNINATIMGGEAGVSYKLTDSWKTDASLAYSWGRNTEDGKPLPQMPPLEARLG-------------
ZnuD                NDGRGPKSIEDDSEMKLVRYNQSGADFYGAEGEIYFKPTPRYRIGVSGDYVRGRL----KNLPSLPGREDAYGNRPFIAQDDQNAP
                                         :  *   *.*    ::     : . :  :  *:     . **.:   *

                             |            |         |         650       |         |            |
P.aeruginosa        --------LTYE--E-GDWSAGSLWRVVAPQNRIARDQGNVVGKDFDKSAGFGVFSLNGAYRVTRNV---KLSAGVDNLFDKDYTE
P.stutzeri          --------LTYE--Q-GDWSAAGLWRVVAAQNRVAEGKGNVTSKDFDESSGFGVFSLNGAYRVNQNF---KLSTGIDNLFDKAYSE
P.putida            --------LTYS--R-DVWSVGALWRLVAAQNRIAENQGNVVGKDYDKSAGFGVFSLNGAYKVNNNL---KLSAGVDNLFDKTYAE
P.syringae          --------LTYE--Q-DTWSAGALWRVVAAQSRVAEGKGNVVGQDFGTSAGFGVFSINGSYKLSKQL---KVSAGVDNLLDKNYAE
A.baumannii         --------IRYV--A-DKYNFGLLWRAVAEQNRVSLHQGNIVGYDLKPSKGFSTLSLNGSYNLRKDI---DVSVGIDNVLDKTYTE
S.enterica          --------LTWD--K-NDWSTTVLWRVVSQQNRIALNEGNVVGKDIIKSPGFTVLSANAAYKFTKDI---KLSIGADNLLNKSYAE
K.Pneumoniae        --------LSWE--S-GNWSSTGLVRLASSQHRVAINEGNVVGKDFDSSAGFAVFSASAAYRVNKYF---KVSAGVDNLLDKDYSE
S.marcescens        --------LTYE--Y-GDWSGTGLWRLVSSQHRVAINEGNVVGKDFAESAGFGVLSANAAYKVNKNV---KLSAGLDNILNKTYSE
E.cloacae           --------LSWE--S-GNWSSTGLIRLVSSQHRVAINEGNVVGKDFDSSAGFAVVSANAAYRVNKYF---KVSAGVDNLLDKDYSE
ZnuD                RVPAARLGFHLKASLTDRIDANLDYYRVFAQNKLARYE--------TRTPGHHMLNLGANYRRNTRYGEWNWYVKADNLLNQSVYA
                            :       .  .       .  * :::  :          : *.  .. .. *.        .     **::::

                             700         |         | 723
P.aeruginosa        HLNKAGDAGFGFSANE--TVPEPGRTFWTKVDFSF
P.stutzeri          HLNQAGNAGIGLSADE--RINEPGRTWWARVDMSF
P.putida            HLNLAGNAGFGYPATDPQPVNEPGRTFWTKVDFSF
P.syringae          HLNLAGDAGFGFAGDK--ALNEPGRTLWTKVDFSF
A.baumannii         HLNKAGSAGFGFASEE--QFNNIGRNYWVRMSMKF
S.enterica          HLNLAGNSGFGYSTDT--IFNEPGRTYWAKLNVVF
K.Pneumoniae        HLNLAGNSSFGYSANT--SVNEPGRTFWGKINVTF
S.marcescens        HLNLAGNSAFGYSANS--AVNEPGRTLWAKVNVTF
E.cloacae           HLNLAGNSSFGYSANT--SVNEPGRTFWGKINVTF
ZnuD                HSS--------FLSD----TPQMGRSFTGGVNVKF
                    * .                  : **.    :.. *
```
