# Supplementary material for: Acquisition of ionic copper by the bacterial outer membrane protein OprC through a novel binding site
Source: PLoS Biol. 2021 Nov 11;19(11):e3001446. doi: 10.1371/journal.pbio.3001446 (PMC8610252; doi:10.1371/journal.pbio.3001446)
Supplement: S1 Table — (DOCX) [file pbio.3001446.s010.docx]

S1 Table. Data collection and refinement statistics for OprC variants with and without copper.

|  | Cu - OprC | OprC_AA_ | Cu – C143A 8800 | Cu – C143A 9175 | H323A – Cu 8800 | H323A – Cu  9175 |
| --- | --- | --- | --- | --- | --- | --- |
| **Data collection**^#^ |  |  |  |  |  |  |
| Space group | C 2 2 21 | P2 21 21 | P 2 21 21 | P 2 21 21 | P 1 21 1 | P 1 21 1 |
| Cell dimensions |  |  |  |  |  |  |
| *a*, *b*, *c* (Å) | 156, 197, 166 | 62, 171, 198 | 171, 198, 67 | 171, 198, 67 | 67,198, 172 | 67, 197, 172 |
| α, β, γ (°) | 90, 90, 90 | 90, 90, 90 | 90, 90, 90 | 90, 90, 90 | 90, 89, 90 | 90, 91, 90 |
| Resolution (Å) | 84.67 – 1.97 | 98.02 – 2.77 | 198.04 -2.78  (2.83 - 2.78) * | 129.57 -2.56 (2.60 - 2.56) * | 197.67 -2.95 (3.00 -2.95) * | 197.27 -2.73 (2.78 -2.73) * |
| *R*_pim_ | 0.062(0.842) | 0.041 (0.748) | 0.078 (1.019) | 0.096 (2.48) | 0.081 (0.554) | 0.056 (0.577) |
| *I* / σ*I* | 15.8 (1.3) | 4.4 (1.6) | 5.52 (0.39) | 5.40 (0.35) | 5.56 (0.58) | 7.82 (0.57) |
| *CC_1/2_* | 0.981 (0.784) | 0.990 (0.56) | 0.994 (0.400) | 0.974 (0.338) | 0.992(0.551) | 0.997 (0.674) |
| Completeness (%) | 99.5(100) | 88.8 (71.1) | 100(100) | 100(100) | 100(100) | 100(100) |
| Redundancy | 25.4 (25.4) | 5.7 (5.5) | 12.7 (12.1) | 12.3(12.3) | 6.6(6.2) | 6.6 (6.4) |
|  |  |  |  |  |  |  |
| **Refinement** |  |  |  |  |  |  |
| Resolution (Å) | 84.67 - 1.97 | 48.67 - 2.9 | 64.66 - 2.78 | 67.02 - 2.56 | 64.81-2.95 | 64.84 - 2.73 |
| No. reflections | 178812 | 42223 | 58104 | 74371 | 93985 | 118703 |
| *R*_work_ / *R*_free_ (%) | 21/ 23.2 | 21.6 / 27.1 | 20.9/26.7 | 24/29.8 | 24.2 /30.3 | 21.7 / 26.7 |
| No. atoms |  |  |  |  |  |  |
| Protein | 10050 | 10058 | 10119 | 10148 | 20154 | 20221 |
| Water | 556 | - | 23 | 39 | - | 67 |
| *B*-factors |  |  |  |  |  |  |
| Protein | 49.27 | 50.16 | 70 | 72 | 67 | 64 |
| Water | 49.28 | - | 54 | 60 | - | 54 |
| R.m.s. deviations |  |  |  |  |  |  |
| Bond lengths (Å) | 0.008 | 0.010 | 0.009 | 0.008 | 0.009 | 0.010 |
| Bond angles (°) | 1.26 | 1.54 | 1.12 | 1.08 | 1.17 | 1.15 |

^#^ One crystal was used for each data collection.

* Values in parentheses are for highest-resolution shell.
