# Supplementary material for: Acquisition of ionic copper by the bacterial outer membrane protein OprC through a novel binding site
Source: PLoS Biol. 2021 Nov 11;19(11):e3001446. doi: 10.1371/journal.pbio.3001446 (PMC8610252; doi:10.1371/journal.pbio.3001446)
Supplement: S2 Table — (DOCX) [file pbio.3001446.s011.docx]

S2 Table. Data collection and refinement statistics for OprC variants with silver.

|  | Ag – OprC  8000 eV | Ag – C143A  8800 eV | Ag – C143A  9175 eV | Ag – H323A  8800 eV | Ag – H323A  9175 eV |
| --- | --- | --- | --- | --- | --- |
| **Data collection**^#^ |  |  |  |  |  |
| Space group | C 2 2 21 | C 2 2 21 | C 2 2 21 | C 2 2 21 | C 2 2 21 |
| Cell dimensions |  |  |  |  |  |
| *a*, *b*, *c* (Å) | 156, 195, 167 | 154, 195, 165 | 155, 196, 166 | 156, 196, 166 | 155,196, 165 |
| α, β, γ (°) | 90, 90, 90 | 90, 90, 90 | 90, 90, 90 | 90, 90, 90 | 90, 90, 90 |
| Resolution (Å) | 121.98 - 2.71  (2.76 - 2.71) * | 165.47 - 2.60  (2.64 - 2.60) * | 84.18 - 2.68  (2.73 - 2.68) * | 70.41 - 2.86  (2.91 - 2.86) * | 63.14 - 2.61  (2.66 - 2.61) * |
| *R*_pim_ | 0.028 (0.503) | 0.055 ( 0.630) | 0.056 (0.654) | 0.207 ( 3.005) | 0.182 (2.940) |
| *I* / σ*I* | 20.12 (1.47) | 7.56 (1.11) | 7.64 (1.09) | 4.18 (0.34) | 5.00 (0.43) |
| *CC_1/2_* | 0.999 (0.653) | 0.992 (0.644) | 0.996 (0.575) | 0.992 (0.284) | 0.993 (0.317) |
| Completeness (%) | 99.9(100) | 99.9(97.6) | 99.9(96.7) | 98.2(97) | 98(96.9) |
| Redundancy | 35.5 (31.6) | 13.5 (13.8) | 13.5(13.9) | 13.1 (13.5) | 13.3(13.6) |
|  |  |  |  |  |  |
| **Refinement** |  |  |  |  |  |
| Resolution (Å) | 97.64 - 2.71 | 82.74 - 2.6 | 82.81 - 2.68 | 70.41 - 2.86 | 60.78 - 2.61 |
| No. reflections | 69472 | 76648 | 70402 | 54313 | 73192 |
| *R*_work_ / *R*_free_ (%) | 21.6 / 25.4 | 22.2 / 26.1 | 21.4 / 25.2 | 20.7 / 27.2 | 21.4 / 27.4 |
| No. atoms |  |  |  |  |  |
| Protein | 10054 | 10156 | 10182 | 10073 | 10112 |
| Water | 13 | 13 | 8 | - | 71 |
| *B*-factors |  |  |  |  |  |
| Protein | 79 | 72 | 72 | 69 | 61 |
| Water | 63 | 57 | 60 | - | 52 |
| R.m.s. deviations |  |  |  |  |  |
| Bond lengths (Å) | 0.008 | 0.008 | 0.009 | 0.009 | 0.008 |
| Bond angles (°) | 1.06 | 1.08 | 1.12 | 1.2 | 1.04 |

^#^ One crystal was used for each data collection.

* Values in parentheses are for highest-resolution shell.
