# Supplementary material for: Acquisition of ionic copper by the bacterial outer membrane protein OprC through a novel binding site
Source: PLoS Biol. 2021 Nov 11;19(11):e3001446. doi: 10.1371/journal.pbio.3001446 (PMC8610252; doi:10.1371/journal.pbio.3001446)
Supplement: S3 Table — (DOCX) [file pbio.3001446.s012.docx]

S3 Table. Data collection and refinement statistics for M147H and M325H variants.

|  | Cu – M147H  9175 eV | Cu – M325H  9175 eV |
| --- | --- | --- |
| **Data collection**^#^ |  |  |
| Space group | C 2 2 21 | C 2 2 21 |
| Cell dimensions |  |  |
| *a*, *b*, *c* (Å) | 155, 197, 165 | 156, 196, 167 |
| α, β, γ (°) | 90, 90, 90 | 90, 90, 90 |
| Resolution (Å) | 98.09 - 2.38 (2.42 - 2.38) * | 97.85 - 2.37 (2.41 - 2.37) * |
| *R*_pim_ | 0.044 (1.056) | 0.044 (1.255) |
| *I* / σ*I* | 11.71 (0.89) | 12.46 (1.00) |
| *CC_1/2_* | 0.996 (0.321) | 0.997(0.315) |
| Completeness (%) | 100 (100) | 100(100) |
| Redundancy | 13.6 (13.2) | 13.3(13.5) |
|  |  |  |
| **Refinement** |  |  |
| Resolution (Å) | 63.34 - 2.38 | 61.02 - 2.37 |
| No. reflections | 101122 | 103039 |
| *R*_work_ / *R*_free_ (%) | 21.8/ 25.3 | 21.9/ 25.2 |
| No. atoms |  |  |
| Protein | 10158 | 10164 |
| Water | 133 | 107 |
| *B*-factors |  |  |
| Protein | 65 | 66 |
| Water | 57 | 28 |
| R.m.s. deviations |  |  |
| Bond lengths (Å) | 0.009 | 0.008 |
| Bond angles (°) | 1 | 0.99 |

^#^ One crystal was used for each data collection.

* Values in parentheses are for highest-resolution shell.
