# Supplementary material for: Acquisition of ionic copper by the bacterial outer membrane protein OprC through a novel binding site
Source: PLoS Biol. 2021 Nov 11;19(11):e3001446. doi: 10.1371/journal.pbio.3001446 (PMC8610252; doi:10.1371/journal.pbio.3001446)
Supplement: Attachment — Submitted filename: Response-to-reviewers.docx [file pbio.3001446.s015.docx]

Dear Editor,

Thank you sending us the constructive comments of the two reviewers for our paper. We have fully addressed their remaining concerns, detailed below. Our responses to the reviewer comments are shown in red font. Likewise, the changes made in the manuscript are also in red font. One unsolicited change we have made is to add a very recent reference (52) that strongly suggests that PA3789 (line 475) is a copper reductase. This supports our notion that OprC transports Cu(II) into the periplasmic space. We have also replaced reference 21 with a more relevant and recent one for Ctr1 structure and function. Finally, we have provided all the source data for our figures in S1_Data.xslx and refer to this at the appropriate places in the text. We hope our manuscript is now acceptable for publication in PLOS Biology.

With best regards,

Bert van den Berg

**Reviewer remarks:**


**Reviewer #1**: Summary: The authors determined the structure of OprC, a TonB-dependent transporter from P. aeruginosa, with and without metal bound. The copper binding site for OprC is unique with the motif CxxxM-HxM motif binding Cu(II) in the structure. The authors also see a "methionine track" leading Cu(I) to the principal metal binding site. Using Ag(I) as a reduced copper mimic, they solved the structure of OprC showing Ag(I) density along the "methionine track". The authors also used EPR and ICP-MS to characterize the specificity to which OprC binds copper. Mutation studies of the residues at the principal binding site further show the specificity of OprC to binding Cu(II) at the binding site, while Cu(I) can be bound to the "methionine track"

Comments:

Figure 1 - It would be helpful if the colors of the protein structures were unique to each structure. Changing the colors of the structures would allow the reader to more easily follow along with the authors' comparison of the structures. Specifically, this will help with 1E and 1F when the authors discuss the differences in the TonB box.

We have left the rainbow scheme for the wild type protein in A and C since this provides useful information. In panels B and D the AA mutant is now shown in cyan, with the plug coloured brown. The same colour scheme for the AA mutant is now used in panels F and I, making it easier to distinguish the two proteins.

Figure 2 - Please include a figure legend for the graphs in Figure 2A-C. This will help the readers follow the data better without having the read the figure captions to understand which samples are being compared.

The panels of Figure 2A-C have been modified to show legends.

The section "OprC is abundant in P. aeruginosa during infection" could be better discussed to explain why the authors performed these experiments.

We have provided some more clarification on why the proteomics were done in lines 393-398:

"Recent studies have suggested that OprC is important for virulence in *P. aeruginosa* and *Acinetobacter baumannii* (37,38). These papers, however, did not provide a measure of the abundance of OprC during infection *in vivo*. We therefore decided to determine the abundance of the highly similar OprC proteins (50% sequence identity, Fig. S7) in *P. aeruginosa* UCBPP-PA14 and *Acinetobacter baumannii* ATCC 19606 in infected lung tissues via a sensitive targeted proteomics approach with parallel reaction monitoring (Methods)."


**Reviewer #2**: This manuscript reports X-ray crystal structures of wild type and mutant OprC, an outer membrane copper importer, in the absence and presence of copper or silver, and characterized metal binding via ICP-MS and EPR. This is a very interesting and thoughtful study that provides detailed structural and mechanistic insights into copper transport by OprC. This is an excellent study shedding light on bacterial copper acquisition. Addressing the following concerns would further increase the significance of this beautiful work.

We thank the reviewer for these comments.

1. Page 9, the OprC methionine track. The authors used silver co-crystal structures to identify 3 distinct binding sites, Ag1, Ag2 and Ag3. Interestingly, 2 binding sites (Ag2 and Ag3) are clearly located in the methionine track. What is the distance between sites Ag2 and Ag3? Could the methionine track simultaneously bind Ag2 and Ag3?

The distance between Ag2 and Ag3 is ~6.5 Å, indicating that two metal ions could likely be bound at the same time. This is probably the case in our structure, given the experimental conditions (~10-fold excess of Ag over OprC). We'd consider it less likely that under physiological conditions two metal ions would be bound simultaneously.

It appears that each site is constructed by 3 methionine residues (which has been seen in other Cu+ transporters) from Figure 3A, with M339 and M343 being shared between these two sites. Please discuss these possibilities.

We now discuss some of these issues in lines 260-270. For example, since the Ag2/Ag3 sites require only five methionines, it is likely that the track (which consists of 11 or 12 methionine residues) forms more than two binding sites. We'd speculate that the sites closer to the irreversible binding site (Ag1) may have a higher affinity, driving the metal towards this site.

2. Transport pathway and mechanism. Very interestingly, Figure 1J reveals the methionine network potentially involved in Cu transport across the outer membrane. The extracellular portion of the Cu path is convincing. Some methionine residues are present in the plug domain. Could the authors outline the Cu path across the membrane? Does the plug domain need to swing out to the periplasm to allow Cu pass through the membrane?

This is an interesting issue, but impossible to answer at this point. What we can say is that there is no channel for substrate diffusion with the plug in place (line 111), so a conformational change of the plug is needed. Another consideration that points to the necessity of a plug conformational change is that metal binding in the principal site (which includes two plug residues) is near-irreversible (lines 190-192). However, the nature of the plug conformational change in TBDTs is still debated. Given the small size of the ligand there seems to be no need for the entire plug to come out of the barrel.

Three of the five "non-track" methionines are roughly on the same side of the barrel as the observed binding sites (panel K). One of these is in the barrel (Met374) and two are in the plug (Met138 and Met139). While these three methionines are close together in the structures, it is unclear whether they could form a metal binding site after TonB-dependent remodelling of the plug. We have added some text to discuss this (lines 276-281) and have labelled these three additional methionines in Fig. 1K.
